# Patient Care and Reciprocal Healing

**DOI:** 10.31486/toj.22.5027

**Published:** 2022

**Authors:** Rev Anthony J. De Conciliis

**Affiliations:** Vice President, Institute of Medicine, Education, and Spirituality at Ochsner, Ochsner Clinic Foundation, New Orleans, LA and Adjunct Professor, The University of Queensland Medical School, Ochsner Clinical School, New Orleans, LA

## TO THE EDITOR

Over the past 10 years, in my role as teacher and consultant at Ochsner Health through the Institute of Medicine, Education, and Spirituality at Ochsner, I have had the privilege to read extensively and develop programs based on the health care literature regarding the character of medicine and, specifically, the care of the patient and the care of the provider. What results from this genuine and caring relationship is reciprocal healing.

I call your attention to a classic article regarding the care of the patient. Ever since its publication in 1927, it has been proven to be timeless in its wise advice. Originally, it was presented as a talk to students at the Harvard Medical School. However, because of its impact, it was published as an article. It continues to be commented on and referred to as an excellent example of value and virtue in medicine today. Indeed, Alton Ochsner, one of the founders of Ochsner Health, adopted many of the author's ideas regarding the comprehensive and humanistic care of patients.

The author was Francis W. Peabody, and his article is entitled “The Care of the Patient.”^1^ It is as relevant today for both students in training and medical professionals in practice as it was for the students who heard it those many years ago. Today, we associate this type of care with words such as reciprocal healing, resilience, integrity, well-being, and patients first. Caring is a mutual experience, so we call it reciprocal. In a letter in the *Ochsner Journal*, the executive committee of the Institute of Medicine, Education, and Spirituality at Ochsner provided a definition of reciprocal healing: “Illness is a multifactorial mosaic experience because it affects the body, mind, and spirit. Any illness has the potential to change the patient, and the healing experience has the potential to change the healer.”^2^ The fact is that caring is always personal and reciprocal.

To give a sample of Peabody's message of care for patients, I quote three passages from the article that indicate the personal nature of a healing relationship.^1^

The treatment of a disease may be entirely impersonal; the care of a patient must be completely personal. The significance of the intimate personal relationship between physician and patient cannot be too strongly emphasized, for in an extraordinarily large number of cases both diagnosis and treatment are directly dependent on it, and the failure of the young physician to establish this relationship accounts for much of his ineffectiveness in the care of patients.

In all your patients whose symptoms are of functional origin, the whole problem of diagnosis and treatment depends on your insight into the patient's character and personal life, and in every case of organic disease there are complex interactions between the pathologic processes and the intellectual processes which you must appreciate and consider if you would be a wise clinician.

The good physician knows his patients through and through, and his knowledge is bought dearly. Time, sympathy, and understanding must be lavishly dispensed, but the reward is to be found in that personal bond which forms the greatest satisfaction of the practice of medicine. One of the essential qualities of the clinician is interest in humanity, for the secret of the care of the patient is in caring for the patient.

I also refer you to one of the many commentaries on the original article, entitled “The Care of the Patient – Francis Peabody Revisited” by Pauline Rabin and David Rabin.^3^ This article opens with the following sentence of praise for the work of Peabody: “This classic essay, with its fabric of pristine humanism, its universality, and its timelessness, embodies the noblest aspirations of the medical profession.”

Perhaps we may reflect on these and other articles that call us to consider once again the essence of caring in the noble profession of medicine. At Ochsner Health, we have established mission and value words that call us to be noble and proclaim in action a character that is firmly based in virtue and values. The contents of Peabody's celebrated article will help all recall a main value in health care—Patients First—a personal aspect of health care. The genuine practice of this value fosters positive qualities of resilience, meaning, and well-being in all health care providers because caring helps to increase reciprocal healing, a bond of virtue between patient and provider. The practice of caring is an indication of true character in medicine and excellent provision of health care.
